# Chronic gastritis

**DOI:** 10.3109/00365521.2015.1019918

**Published:** 2015-04-22

**Authors:** Pentti Sipponen, Heidi-Ingrid Maaroos

**Affiliations:** ^a^ Patolab Oy, Espoo, Finland and Tartu State University, Tartu, Estonia

**Keywords:** gastric cancer, gastritis, *Helicobacter pylori*, peptic ulcer

## Abstract

Prevalence of chronic gastritis has markedly declined in developed populations during the past decades. However, chronic gastritis is still one of the most common serious pandemic infections with such severe killing sequelae as peptic ulcer or gastric cancer. Globally, on average, even more than half of people may have a chronic gastritis at present. *Helicobacter* *pylori* infection in childhood is the main cause of chronic gastritis, which microbial origin is the key for the understanding of the bizarre epidemiology and course of the disease. A life-long and aggressive inflammation in gastritis results in destruction (atrophic gastritis) of stomach mucosa with time (years and decades). The progressive worsening of atrophic gastritis results subsequently in dysfunctions of stomach mucosa. Atrophic gastritis will finally end up in a permanently acid-free stomach in the most extreme cases. Severe atrophic gastritis and acid-free stomach are the highest independent risk conditions for gastric cancer known so far. In addition to the risks of malignancy and peptic ulcer, acid-free stomach and severe forms of atrophic gastritis may associate with failures in absorption of essential vitamins, like vitamin B12, micronutrients (like iron, calcium, magnesium and zinc), diet and medicines.

## Introduction

Chronic gastritis is one of the most common life-long, serious and insidious illnesses in human beings. One may estimate that more than half of the world population have this disease in some degree and extent, indicating that even many hundreds of millions of people worldwide may have chronic gastritis in a form or other.

The significance of chronic gastritis as a serious disease is largely underrated in clinical practice, even though the role of gastritis in the pathogenesis of ordinary peptic ulcers and gastric cancers is obvious [1, 2, 3, 4]. One may estimate that millions of premature deaths may occur annually worldwide due to cancer and ulcer as sequelae of the chronic gastritis.

Chronic gastritis appears either as nonatrophic or atrophic form. They are forms and phenotypes of gastritis which represent different stages of a same life-long disease [2, 3, 5, 6, 7]. The morphological appearances of gastritis published are very similar worldwide, i.e., chronic gastritis is seemingly, with its sequelae, one and same disorder throughout the world.

Chronic gastritis has been known and studied since the early decades of the 20th century but received more attention not until 1982 after discovery of the *Helicobacter pylori* by Warren and Marshall [8]. It has become clear that the bacterium is the cause of gastritis in an overwhelming majority of the cases, a possible exception being a gastritis of the autoimmune origin [3, 9]. Consequently, it has become evident that chronic gastritis can be cured with eradication of *H. pylori*, resulting in normalization of the gastric mucosa, at least in cases in which the gastritis is not developed to atrophic (atrophic gastritis) end stages [8, 9, 10, 11, 12, 13].

Even though the main outlines of chronic gastritis are well known, several unanswered questions occur still. We do not know, for example, the significance of autoimmunity or genetics in the development and progression of chronic *H. pylori* gastritis. The molecular mechanisms and the role of environmental factors, like diet, and the role of other microbes than *H. pylori* on the course of chronic gastritis, are largely unknown. We cannot precisely predict either in whom the chronic gastritis will certainly progress to atrophic end stages and to killing sequelae, or in whom it will not. This uncertainty is also the case regarding the details by which the gastritis accomplishes the appearances of peptic ulcers or gastric cancer. We know, however, that without the presence of a coexisting chronic gastritis or atrophic gastritis, ordinary peptic ulcers or stomach cancers are rare.

This review describes some observations and presents some subjective opinions and remarks on course and epidemiology of chronic *H. pylori* gastritis and related diseases obtained mainly from several studies in Estonia and Finland during the past 40 years [14, 15, 16, 17, 18, 19, 20, 21, 22]. Over the years, most of these studies have been published in this journal.

## Natural course

**Figure 1.**
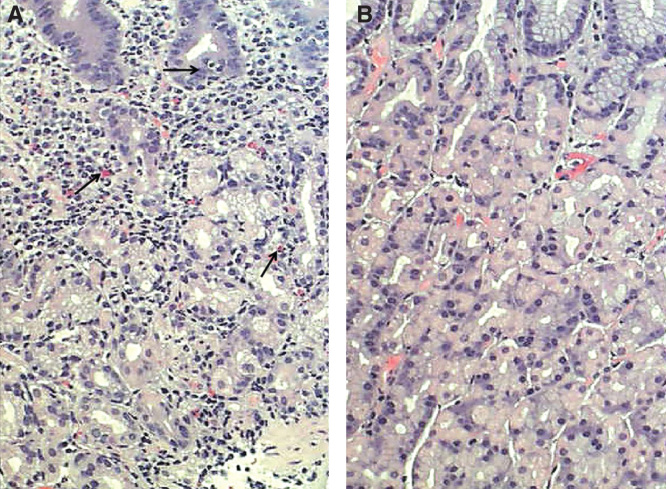
**Corpus mucosa with “active” chronic gastritis (A): Inflammation is mononuclear but is accompanied with neutrophils and eosinophils (arrows) which penetrate into the surface epithelium. Normal (B): Normal corpus mucosa without any sign of inflammation is shown as a reference. HE stain × 500.**

**Figure 2.**
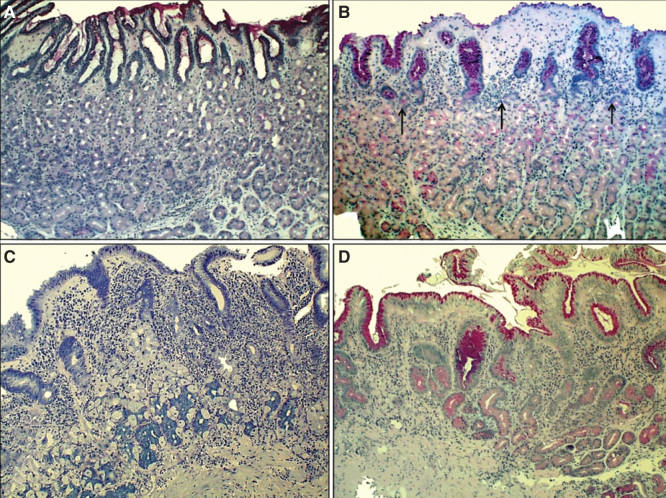
**Corpus mucosa. Normal (A): The layer of oxyntic glands is normal suggesting that the corpus mucosa is capable to secrete hydrochloric acid normally. Non-atrophic gastritis (B): Mild mononuclear inflammation is seen in upper layer (foveolar part) of the mucosa (“superficial chronic gastritis”) as indicated with arrows. The gland layer is intact suggesting that the acid secretion is normal in spite of the gastritis. Moderate atrophic gastritis in corpus (C): Intense chronic mononuclear inflammation occurs also in lower layers of the mucosa and is accompanied with a marked loss (atrophy) of normal oxyntic glands. The observation suggests that the stomach is hypochlorhydric but is not achlorhydric. Acid secretion is impaired due to loss of the parietal cells. Severe atrophic gastritis in corpus (D): Chronic inflammation is mild but all oxyntic glands are totally gone. Some foci of intestinal metaplasia occur in the lower right corner. Stomach is certainly acid free (achlorhydric). The patient is at risk for malabsorption of vitamin B12, and also the absorption of micronutrients (iron, calcium, magnesium and zinc) may be impaired. The pathologist may have difficulties to find *Helicobacter pylori* organisms in cases like this even though the atrophic gastritis would be of *H. pylori* origin. Instead, a mixed microbial flora (microbes other than *H. pylori*) is a common finding on the surface mucosa in cases like this. Alcian blue – PAS and modified Giemsa stains × 300.**

**Figure 3.**
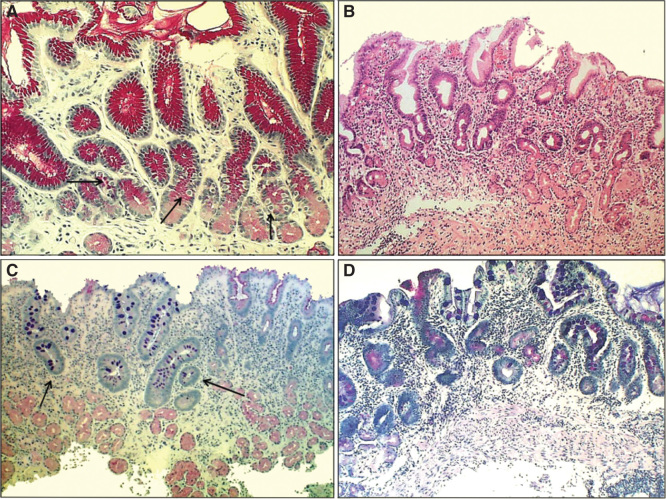
**Antral mucosa. Normal (A): Normal number of antral G cells with typical “hallo” appearances occur in neck area of the pyloric glands (arrows). Non-atrophic chronic gastritis (B): Mononuclear inflammation occupies the whole mucosa giving an impression of gland loss (atrophy). Atrophic gastritis of mild to moderate degree in antrum (C): Inflammation is relative mild but there is a large area of pyloric glands lost and replaced with metaplastic glands (IM) as indicated with arrows. Severe atrophic gastritis in antrum (D): All pyloric glands are gone and the whole mucosa is “intestinalized”. Inflammation is mild, or moderate at most. Also the antral G cells are disappeared along with the loss of the normal pyloric glands. Therefore, the G-17 feedback response in physiological control of acid secretion is impaired. HE and Alcian blue – PAS stains × 300.**

**Figure 4.**
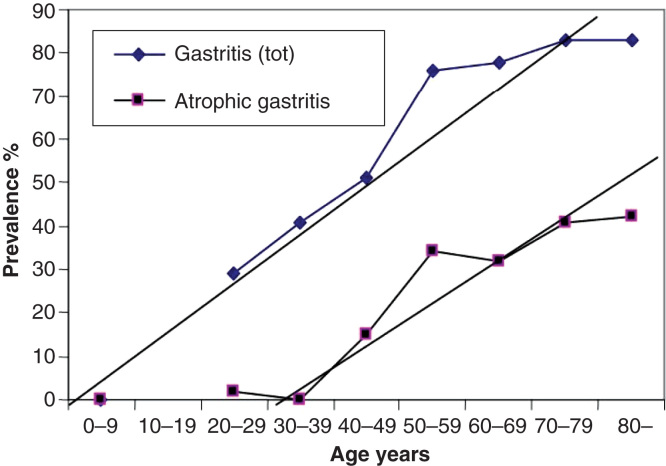
**Mean age-specific prevalence of total chronic gastritis and atrophic gastritis in biopsy samples from ∼500 consecutive endoscopy Finnish outpatients in the late 80s (Jorvi hospital, Espoo, Finland). The mean prevalences increase with age.**

Chronic gastritis is a multistep, progressive and life-long inflammation [3, 16, 20, 21, 22, 23, 24]. It begins usually in childhood as a simple chronic (“superficial”) mononuclear inflammation with co-existence of an acute (“active”) neutrophilic inflammation of varying degree [25, 26]. Gastritis progresses stepwise, within years and decades, to atrophic gastritis that is characterized by a loss of normal mucosal glands either in antrum or corpus (and fundus), or in both (Figures 1, 2 and 3) [7, 22, 23, 27]. Typically in all populations, the age-specific prevalence of both non-atrophic and atrophic gastritis tends to rise by age as is demonstrated from Finland in Figure 4. At present, the increase of the age-specific prevalences of gastritis by age is more pronounced and abrupt in the developing than in the developed populations, i.e., the prevalence of gastritis in young age-groups, or even in childhood, in much more than 50% in developing populations, whereas this prevalence in developed population is typically much less than 50% [17, 27].

The loss of mucosal glands in atrophic gastritis is replaced with a growth of new immature glandular and epithelial elements; that is, with glands of intestinal type (“intestinal metaplasia (IM)”), resembling glands and epithelium in colon and/or small bowel, and/or with pyloric type (“pseudopyloric metaplasia”), resembling pyloric glands and epithelium from which the G cell (gastrin cell) are disappeared. In frames of the evolution, the highly differentiated glands, epithelium and cells are destroyed in atrophy (atrophic gastritis) and the lost glands are replaced by glands and epithelium with immature intestinal properties [2, 5, 6, 7, 14, 17, 23, 24, 27, 28]. Figures 2 and 3 give some examples of these phenomena in endoscopic biopsy pathology.

The first appearances of gastritis of the *H. pylori* etiology tend to be antral, i.e., gastritis is “antral predominant” [25, 27]. The inflammation, composing mainly of mononuclear inflammatory cells and plasma cells, is “superficial” and occupies the upper layers of the mucosa, pronouncedly in corpus (Figure 2) [2, 20, 27, 28, 29]. The chronic inflammation associates with a neutrophilic inflammation (Figure 1), the intensity of this acute and “active” component of gastritis being most likely dependent on the cytotoxicity of the *H. pylori* strain [22, 30]. The more cytotoxic the strain is, the more active, and, obviously, more aggressive is the chronic gastritis. The most aggressive forms of chronic gastritis are those which result most likely in advanced stages of atrophic gastritis, i.e., are forms of *H. pylori* gastritis with highest likelihood to progress to the end-stage atrophy [5, 6].

The annual risk of progression of chronic gastritis from one step to the next one is estimated to be 2–3% on average [16, 20, 23, 27]. It is estimated further that ∼50% of patients with chronic gastritis (and *H. pylori* infection) will get atrophic gastritis of some grade and extent during the life-time [27]. In some 5% of the infected people, atrophic gastritis will get a severe and advanced stage [23, 27].

A slow extension of *H. pylori* gastritis from a pure antral phenotype to forms that affect also the corpus and fundus (“atrophic pangastritis”, multifocal atrophic gastritis” or “corpus predominant gastritis”) tends to be a common path [7, 15, 19, 21, 23, 27, 31]. This proximal (pyloro-cardial) spread and the gradual worsening of atrophic gastritis associate with marked changes in morphology and function of the stomach mucosa. The affections interfere stomach functions by causing particularly failures in secretion of hydrochloric acid and intrinsic factor from oxyntic glands in corpus atrophy, and failures in synthesis and secretion of gastrin-17 from antral G cells in antral atrophy [23, 27, 32, 33, 34].

A severe atrophic gastritis that is strictly limited to antrum alone is obviously quite rare. On the other hand, an advanced atrophy that occurs in corpus alone, or occupies both antral and corpus compartments simultaneously, is a relatively common phenotype of chronic gastritis (in up to 5% of people with gastritis), in the Northern Europe at least [27, 31, 35]. The long-term follow-up studies suggest that, along with the upward extension of *H. pylori* gastritis, the antral mucosa may even heal [20, 23, 27].

Due to the loss of oxyntic glands and parietal cells in corpus atrophy, the extension of atrophic gastritis to corpus and fundus results in a life-long and permanent hypo- or achlorhydria. Subsequently, due to a low intragastric acidity, the initial *H. pylori* infection may, in turn, spontaneously fade and may finally heal in cases with an advanced atrophy as is demonstrated with serology tests in the follow-ups [29, 36].

**Figure 5.**
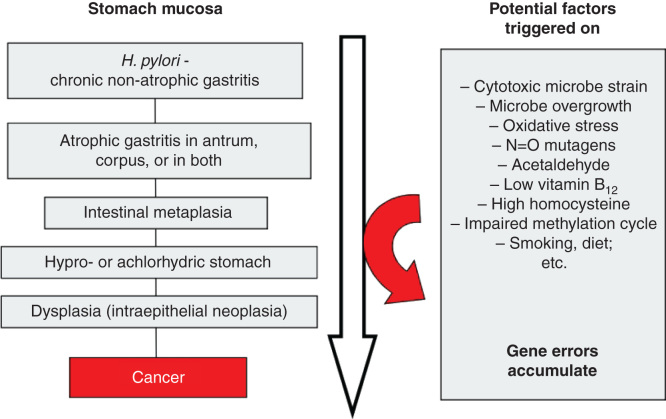
**Scheme on natural course and progression of *Helicobacter pylori* gastritis from a non-atrophic form to gastric malignancy (sc. “Correa cascade”). Several potentially pathogenetic factors and mechanisms, linked with carcinogenesis, play a role and are triggered stepwise on during the course and progression of the cascade.**

Along the progression of chronic non-atrophic gastritis to atrophic phenotypes, manifold coincidental pathogenetic processes, even carcinogenic ones, are potentially triggered on, which phenomena in sc. “Correa cascade” may finally contribute to processes that link the chronic gastritis with such extreme sequelae as cancer [1, 7, 35, 37, 38, 39, 40, 41]. This can be exemplified as illustrated in Figure 5.

## Epidemiology and the birth cohort phenomenon

The observations from population studies on chronic gastritis may empower some fundamental conclusions concerning the epidemiology of chronic gastritis and its sequelae.

Chronic gastritis is still a relatively common disease, also in developed countries even though its prevalence has markedly declined, this decline being parallel with a decline of the *H. pylori* prevalence [42, 43]. It is conceivable that practically everyone, even in developed countries, had *H. pylori* gastritis some 100 years ago, whereas the prevalence may be now less than 50% on average.

*H. pylori* gastritis is acquired in early childhood in most of the cases, the phenotype of gastritis being then a harmless and symptom-free simple chronic mononuclear (“superficial”) inflammation [25, 29]. The severe symptomatic sequelae of chronic gastritis will appear not until in last decades of the life, and tend arise in subjects with the advanced end stages lesions [1, 39, 43].

After discovery of *H. pylori*, it became evident that the understanding of the epidemiology of *H. pylori* infection is the key for the understanding of the epidemiology of chronic gastritis and its sequelae as well. As being initially a pandemic pediatric bacterial infection, the epidemiological characteristics of chronic gastritis and its sequelae (peptic ulcer and gastric cancer) follow the same “pandemic” principles as those of *H. pylori* infection by itself.

Socioeconomics and environmental hygiene are inevitably the most important background factors in transmission of *H. pylori* infection worldwide, these socioeconomic factors being, thereby, the background factors also in epidemiology of chronic gastritis and its sequelae [43, 44, 45]. Socioeconomic status in childhood, environmental and family-bound hygiene, household density, cooking habits, etc., are plausible factors that determine the likelihood to acquire the *H. pylori* infection in the childhood. Improvements in these conditions diminish the likelihood to get the infection, or to transmit the infection further, by a gastro-oral route most likely [46]. A decline of the infection rate in childhood at the family level will inevitably result, with time, in a decline of the prevalence of both *H. pylori* gastritis and its sequelae also in the whole population on average.

**Figure 6.**
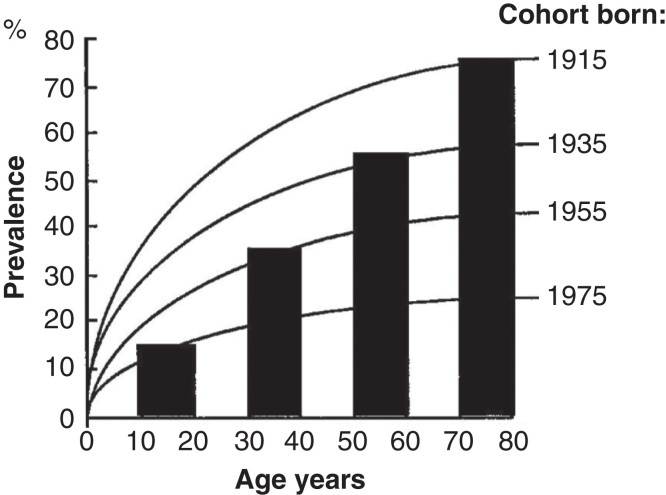
**A computer simulation to demonstrate the “birth cohort phenomenon” in prevalence of *Helicobacter pylori* gastritis in a population consisting of cohorts with dissimilar birth years. Approximated from data on gastritis in Finland around the late 80s (see Figure 4).**

Due to the “pediatric” origin of *H. pylori* infection, the epidemiology of *H. pylori* gastritis is best figured out and understood as a “birth-cohort phenomenon” as is exemplified with a computer simulation in Figure 6 [43]. The infection rate in childhood and the age-specific prevalence of *H. pylori* gastritis are high in the “old” birth cohorts born decades earlier than the prevalence in the “young” birth cohorts born more recently and in whom the infection rate of *H. pylori* at childhood is low. Thus, the mean prevalence of gastritis at the population level reflects the average of the prevalences of chronic gastritis in different birth cohorts, and the mean rate of *H. pylori* infection at pediatric age.

The epidemiology of stomach cancer incidence, and the epidemiology of stomach cancer mortality, follow the principles of the “birth cohort phenomenon” and show similar global “pandemic” characteristics as the case is with the *H. pylori* gastritis [43]. The incidence rate of gastric cancer (or peptic ulcer) is and will be low in the “young” birth cohorts in whom the incidence of *H. pylori* gastritis is low and in whom the stomach mucosa remains healthy through the whole life-span. Instead, the prevalence, incidence and mortality for peptic ulcer or gastric cancer are high, and remain high, in the old cohorts as long as the cohorts live.

## Autoimmune chronic gastritis

Although *H. pylori* is ultimately the major cause (in more than 90% of the cases) of gastritis, a chronic mononuclear inflammation (gastritis) without an on-going *H. pylori* infection, but occurring with a severe atrophic corpus gastritis and achlorhydric stomach, and with co-existence of auto-antibodies against parietal cells (proton pump) and/or intrinsic factor, is a well established entity [3, 32, 47, 48, 49, 50]. It is uncertain, however, whether this gastritis of “autoimmune” type is an independent disorder or whether the *H. pylori* triggers autoimmune reactions on in some liable individuals with an ordinary *H. pylori* infection [3, 50, 51, 52]. It is possible that the initial *H. pylori* infection may pass spontaneously out when the atrophic gastritis is developed to severely atrophic and achlorhydric stage, giving, thereby, a false impression of a pure autoimmune etiology of the disease.

A gastritis linked with the autoimmune phenomena may anyhow be more progressive and faster in course than the pure *H. pylori* gastritis [3, 23]. In connection with autoimmunity, and in persons with a liability to autoimmune diseases (autoimmune thyreoiditis, diabetes, etc.), the progression of *H. pylori* gastritis to atrophic phenotypes may be markedly destructive, possibly resulting easily and quickly in achlorhydria and in total atrophy of gastric corpus and fundus. Cases with severe atrophic corpus gastritis with autoimmune characteristics and with acid-free stomach even in childhood have been described in the medical literature.

Chronic gastritis of the autoimmune type is shown to be a recessive multigenic disease and is possibly of Northern European heritage [32].

## Influences on stomach physiology

A long-lasting (life-long) chronic and active inflammation cannot be harmless and will result in destruction of stomach mucosa *via* several mechanisms which may interact with renewal, growth, integrity, differentiation and function of the gastric epithelium [1, 24, 39, 53, 54]. Numerous abnormal expressions (up- and down-regulations) of functional and regulatory genes, gene mutations, epigenetic alterations, variable appearances of cytokines or tissue growth factors occur in the regulatory tissue processes of gastric mucosa and epithelial cells in connection with *H. pylori* gastritis [53, 54]. Some of the alterations are reversal, may appear even at early stages of inflammation, but may particularly accumulate with time in premalignant conditions, such as atrophy and IM, and seem to be extremely common and manifold in precancerous (dysplasia or intraepithelial neoplasia) and malignant lesions [53, 54].

Corpus atrophy leads ultimately to failures in the secretion of hydrochloric acid and intrinsic factor [23, 34]. In acid-free and atrophic stomach, due to the impairment in secretion of intrinsic factor, absorption of the essential vitamins, like vitamin B12, are severely failed [55]. Malabsorption and subsequent long-lasting and permanent deficiency of vitamin B12 can impair the metabolism of methionine, homocysteine or folate, and may be, thereby, a mechanism which contributes to the appearances of epigenetic DNA damages via impairments in function of the methylation cycle in epithelial cells [55, 56].

Dietary metabolism and absorption of micronutrients, like iron, calcium, magnesium and zinc, or absorption of some medicines (e.g., dipyridamol, some iron formulations and anti-fungal medicines like fluconazole or itraconazole, thyroxin and atazanovir) are impaired in acid-free stomach too [57]. The acid-free stomach is ultimately colonized with a microbe flora from the mouth [58]. The microbes and the acid-free stomach are capable to produce class 1 carcinogens, like acetaldehyde and nitrosoamines [39, 59].

## Cancer and peptic ulcer

Gastric cancer and peptic ulcer diseases (excluding NSAID ulcers) are the most serious diseases linked with the chronic *H. pylori* gastritis [60, 61, 62, 63, 64, 65]. A successful eradication of the *H. pylori* prevents ulcer recurrences but may also prevent cancers [66, 67].

**Figure 7.**
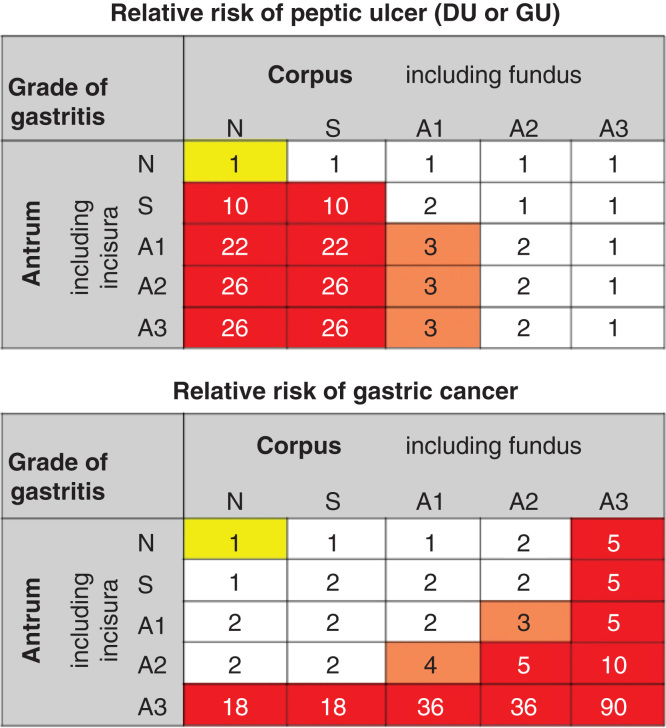
**Relative risk of peptic ulcer disease (duodenal or gastric ulcer) or gastric cancer in various phenotypes of chronic atrophic *Helicobacter pylori* gastritis. The risks are presented as relative risks compared to risks in subjects with normal and healthy stomach mucosa (N/N category; yellow). The risks are extrapolated and estimated from a case-control study done in Finland in the 80s [69]. Note that the risks of peptic ulcer diseases and gastric cancer are associated with very dissimilar phenotypes of chronic gastritis.**

In spite of the associations of peptic ulcers and cancer with *H. pylori* gastritis, there also are remarkable dissimilarities regarding these links [62, 68]. Peptic duodenal ulcers (DU) occur typically in people with non-atrophic *H. pylori* gastritis but are infrequent or absent in subjects in whom the gastritis has progressed to atrophic forms in corpus, and in whom the stomach is, subsequently, hypochlorhydric (Figure 7). Gastric ulcers associate often, on the other hand, with the phenotypes of gastritis in which a marked atrophy and IM occur in antrum and incisura, and in which cases, the corpus mucosa may even be slightly atrophic but is never severely atrophic (and stomach is never achlorhydric) [62]. The stomach cancer, the intestinal type in particular, is seen in patients with a hypochlorhydric or achlorhydric stomach and may particularly occur in subjects with advanced stages of atrophy and IM in both antrum and corpus [39, 69].

Gastric carcinogenesis is heterogeneous and extremely varied at the molecular level [53, 54]. Even though the gene errors are plentiful, no specific or comprehensive molecular markers, gene errors or mutations have been found in cancer or in precancer lesions so far. Although the two main histological types of stomach cancer, intestinal and diffuse one, are meaningful entities in clinical and morphological perspectives, and both being bound to a preceding or co-existing *H. pylori* gastritis, the types may not be fully distinct entities. In diffuse type, the background morphology is often a non-atrophic *H. pylori* gastritis, indicating that the carcinogenetic mechanisms in this cancer type may be linked more closely with the inflammation than are associated with the mechanisms that appear in atrophic gastritis or acid free stomach [37, 38]. In intestinal cancer type, on the other hand, the overt tumors or the precancer lesions associate often with a hypochlorhydric stomach, and with a marked atrophic gastritis, and with extensive IM in the underlying mucosa [37, 38, 70, 71]. Therefore, the carcinogenetic processes in this cancer type may be linked with mechanisms that arise in atrophic gastritis or are triggered on by a hypochlorhydric or acid-free stomach.

## Cancer risk

Atrophic gastritis and acid-free stomach are the most important independent risk conditions for stomach cancer known so far. Statistically, the atrophic gastritis is a higher risk condition for cancer than the pure *H. pylori* infection [69]. The likelihood of gastric cancer in gastritis rises exponentially with the progression of the *H. pylori* gastritis from a non-atrophic form to an atrophic form [69]. The risk may rise even to 90-fold in patients with severe atrophic gastritis in both antrum and corpus (severe panatrophy; multifocal atrophic gastritis (MAG)) compared to the cancer risk in subjects with a normal and healthy stomach mucosa (Figure 7) [69].

The cancer risk is remarkably low in people without the *H. pylori* gastritis, i.e., in subjects with a healthy stomach mucosa. The risk rises approximately to two-fold in patients with a pure *H. pylori* infection, i.e., in subjects with a non-atrophic *H. pylori* gastritis [60, 61, 69]. Thus, in DU patients, who typically have the non-atrophic *H. pylori* gastritis, the cancer risk is slightly higher than the cancer risk in healthy subjects, but this risk is still, however, remarkably low as compared to the cancer risk in patients with a severe and advanced atrophic gastritis (see Figure 7).

Paradoxically, the load of the stomach mucosa with helicobacteria is typically high in subjects with a relatively low cancer risk, as the case is in the ordinary DU patients. Paradoxically again, the load tends to diminish when the cancer risk rises, as the case is in the patients with advanced atrophic gastritis [69]. The cancer risk may be, for example, high in subjects with a severe atrophic gastritis and acid-free stomach in whom there may even be no objective signs of an on-going *H. pylori* infection, i.e., in patients in whom the initial *H. pylori* infection is possibly passed out.

## The Sydney system and operative link on gastritis classifications

The acceptance that *H. pylori* infection and subsequent atrophic gastritis, and acid-free stomach, are important background conditions of gastric cancer enables the sorting of patients in clinical practice to meaningful groups regarding the cancer risk. This sorting requires examination of the subjects with endoscopy and endoscopy biopsy, or can be done with non-invasive blood tests applying the stomach-specific biomarkers [72, 73, 74, 75, 76, 77].

The Sydney System and its updating are the guidelines for interpretation of the microscopical appearances in biopsy specimens in endoscopy practice [5, 6]. A proper biopsy protocol and correct microscopical reading of the biopsies are fundamental requirements for a correct and successful delineation of patients into the clinically meaningful risk groups [78, 79]. Such classifications or lineations are not possibly with endoscopy alone, or cannot be done with testing of the *H. pylori* alone.

**Figure 8.**
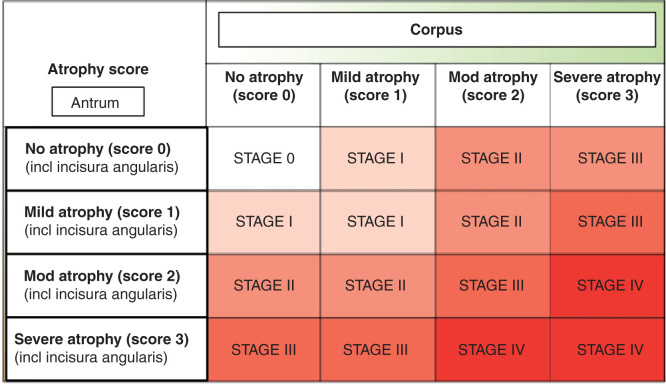
**OLGA staging for risk of stomach cancer. Modified from a paper of Rugge et al [79]. The staging is a practical tool for the delineation of patients to high (stages III–IV) and low (stages 0–II) risk groups for cancer, for gastric cancer of the intestinal type in particular. The staging requires endoscopy and proper biopsy practice but can also be done non-invasively by a blood test with applying specific biomarkers (pepsinogen I and II, gastrin-17 and *Helicobacter pylori* serology) that reflect the function (acid secretion) and the structure of both antral and corpus mucosa in the blood plasma/serum.**

The OLGA/OLGIM staging (Operative Link on Gastritis/IM Assessment) of atrophic gastritis is a practical scheme for the categorization of the patients to low-, medium- and high-risk groups for stomach cancer [79]. This classification delineates the patients into five subgroups (0–IV) with markedly dissimilar likelihood to have or get a gastric cancer (Figure 8). The classification and sorting may objectively advice which one of the patients would need and benefit most of endoscopic surveillances and follow-ups [79, 80].

The delineation of the subjects into OLGA subcategories can be performed also non-endoscopically and noninvasively by applying simple blood tests, based on assessment of the levels of the stomach-specific biomarkers (pepsinogens I and II, gastrin-17 and *H. pylori* antibodies) in blood plasma/serum [72, 73, 74, 77, 80]. This cannot be done with the *H. pylori* tests alone.

## Plasma biomarkers in diagnosis and screening of atrophic gastritis

Purification of stomach pepsinogens in the 70s, understanding of natural course of *H. pylori* infection, unique innovations in microwell plate vertical measurement techniques and in development of ELISA tests with specific monoclonal antibodies have provided applications to use gastric biomarkers in non-invasive diagnosis and screening of atrophic gastritis from a simple blood sample [81, 82, 83]. At present, these blood tests can be performed reliably in any laboratory and they do not need special arrangements. Several detailed and comprehensive reviews, consensus reports and guidelines on practical usage of the biomarker tests in the diagnosis of atrophic gastritis have been published previously in this and other medical journals (see references [72, 80, 84]).

Several commercial radioimmunoassays and ELISA tests have been available for pepsinogen 1 and 2 for a quite long time. These tests have, however, limitations because they can be used in the diagnosis of atrophic corpus gastritis only [74]. The serum levels of pepsinogens 1 and 2 do not give any hints of possible atrophic gastritis in gastric antrum where the atrophic and metaplastic alterations appear, however, first. A more complete test (GastroPanel®) includes an assay of the plasma levels of G-17, in addition to the assays of pepsinogen 1 and pepsinogen 2, and includes also the *H. pylori* serology test. This test panel enables assessments of atrophy also in the gastric antrum and gives hints of intragastric acidity in addition to the information of possible corpus atrophy, and makes, therefore, the comprehensive OLGA classification possible [72, 73, 77, 78, 80].

Antral atrophic gastritis (loss of antral glands) is accompanied with a loss of antral G cell, which cell loss results in low plasma levels of both fasting and stimulated gastrin-17 (after a drink with protein powder, stimulation with bombesin (gastrin releasing peptide) or stimulation with proton pump inhibitor (PPI)). A low fasting plasma G-17 (1 pmol/l or less) indicates two options in practice. Either there is an advanced atrophic gastritis in the antrum (OLGA class III–IV) or the low G-17 is a result of a high intragastric acidity (low pH). Both options are indications for upper gi-endoscopy because of the cancer risk due to possible antral atrophy and because of the peptic ulcer risk due to hyperchlorhydria (high-acid output) (see Figure 7).

The absence of *H. pylori* antibodies concomitantly with the presence of normal plasma levels of pepsinogen 1, with normal ratio of pepsinogen 1 to pepsinogen 2, and with normal plasma gastrin-17, providing that a possible PPI medication is controlled, is a test result that reliably suggests the presence of normal and healthy stomach mucosa (OLGA class 0). The risk of cancer and peptic ulcer in such “healthy” subjects is extremely low, practically nil. The risk of gastric cancer is, on the other hand, high (OLGA class III-IV) in subjects with low levels of fasting pepsinogen 1 (less than 30 microg/l), with low pepsinogen 1 to pepsinogen 2 ratio (lower than 3) and with high fasting gastrin-17 (above 7 pmol/l), The cancer risk is high in these patients irrespectively of whether the patient has or does not have an on-going *H. pylori* infection (see Figure 8) [72, 80]. The atrophic gastritis is advanced and extensive in these cases, occupies the whole stomach mucosa (OLGA class IV), and the stomach is concomitantly acid free.

## Future trends and the healthy stomach initiative

Even though the *H. pylori* gastritis is declining in prevalence, chronic gastritis will still be a remarkably frequent disease globally for several future decades. Due to the microbial origin and infectious background, and by the knowledges of epidemiology and natural course of chronic *H. pylori* gastritis, one can assume that this disease may finally disappear from the society, along with the improvements in hygiene and in socioeconomics. With these improvements, the risk to acquire *H. pylori* infection in childhood will decline. As the case is with tuberculosis, chronic gastritis may move in the future decades to periphery of the medical practice and significance, in developed countries at least. Consequently, peptic ulcers will be rare and gastric cancer will become an infrequent malignancy.

In the youngest native citizens in birth cohorts in western countries, some 90–100% of people already now have and will have a normal healthy stomach for their whole life-span, resulting finally in populations in which the exceeding majority of people have a healthy stomach and are, therefore, free of risks of peptic ulcer or stomach cancer. The attempts to maintain or to receive a healthy stomach to everybody will be an initiative and a goal in both future research and clinical practice (“healthy stomach initiative”; see: www.hsinitiative.org).

## References

[CIT0001] IARC (1994). Schistosomes, liver flukes and Helicobacter pylori. IARC Working Group on the Evaluation of Carcinogenic Risks to Humans. Lyon, 7-14 June 1994. IARC Monogr Eval Carcinog Risks Hum.

[CIT0002] Schindler R (1966). Chronic gastritis. Klin Wochenschr.

[CIT0003] Siurala M (1991). The story of gastritis. Scand J Gastroenterol Suppl.

[CIT0004] Telaranta-Keerie A, Kara R, Paloheimo L, Härkönen M, Sipponen P (2010). Prevalence of undiagnosed advanced atrophic corpus gastritis in Finland: an observational study among 4,256 volunteers without specific complaints. Scand J Gastroenterol.

[CIT0005] Price AB (1991). The Sydney System: histological division. J Gastroenterol Hepatol.

[CIT0006] Dixon MF, Genta RM, Yardley JH, Correa P (1996). Classification and grading of gastritis. The updated Sydney System. International Workshop on the Histopathology of Gastritis, Houston 1994. Am J Surg Pathol.

[CIT0007] Correa P (1988). Chronic gastritis: a clinico-pathological classification. Am J Gastroenterol.

[CIT0008] Marshall BJ, Warren JR (1984). Unidentified curved bacilli in the stomach of patients with gastritis and peptic ulceration. Lancet.

[CIT0009] Varis K (1981). Family of behavior of chronic gastritis. Ann Clin Res.

[CIT0010] Valle J, Seppälä K, Sipponen P, Kosunen T (1991). Disappearance of gastritis after eradication of Helicobacter pylori. A morphometric study. Scand J Gastroenterol.

[CIT0011] Hojo M, Miwa H, Ohkusa T, Ohkura R, Kurosawa A, Sato N (2002). Alteration of histological gastritis after cure of Helicobacter pylori infection. Aliment Pharmacol Ther.

[CIT0012] Annibale B, Di Giulio E, Caruana P, Lahner E, Capurso G, Bordi C (2002). The long-term effects of cure of Helicobacter pylori infection on patients with atrophic body gastritis. Aliment Pharmacol Ther.

[CIT0013] Arkkila PE, Seppälä K, Färkkilä MA, Veijola L, Sipponen P (2006). Helicobacter pylori eradication in the healing of atrophic gastritis: a one-year prospective study. Scand J Gastroenterol.

[CIT0014] Siurala M, Varis K, Wiljasalo M (1966). Studies of patients with atrophic gastritis: a 10–15-year follow-up. Scand J Gastroenterol.

[CIT0015] Villako K, Kekki M, Tamm A, Tammur R, Savisaar E, Viirsalu V (1982). Epidemiology and dynamics of gastritis in a representative sample of an Estonian urban population. Scand J Gastroenterol.

[CIT0016] Ihamäki T, Kekki M, Sipponen P, Siurala M (1985). The sequelae and course of chronic gastritis during a 30- to 34-year bioptic follow-up study. Scand J Gastroenterol.

[CIT0017] Maaroos HI, Villako KP, Sipponen P, Kekki M, Siurala M, Tammur R (1990). Helicobacter pylori and chronic gastritis in the gastric biopsy material in a group of randomly selected adult inhabitants of Estonia. Arkh Patol.

[CIT0018] Kekki M, Maaroos HI, Sipponen P, Uibo R, Tammur R, Tamm A (1991). Grade of Helicobacter pylori colonization in relation to gastritis: a six-year population-based follow-up study. Scand J Gastroenterol Suppl.

[CIT0019] Villako K, Kekki M, Maaroos HI, Sipponen P, Uibo R, Tammur R (1991). Chronic gastritis: progression of inflammation and atrophy in a six-year endoscopic follow-up of a random sample of 142 Estonian urban subjects. Scand J Gastroenterol Suppl.

[CIT0020] Valle J, Kekki M, Sipponen P, Ihamäki T, Siurala M (1996). Long-term course and consequences of Helicobacter pylori gastritis. Results of a 32-year follow-up study. Scand J Gastroenterol.

[CIT0021] Villako K, Kekki M, Maaroos HI, Sipponen P, Tammur R, Tamm A (1995). A 12-year follow-up study of chronic gastritis and Helicobacter pylori in a population-based random sample. Scand J Gastroenterol.

[CIT0022] Maaroos HI, Vorobjova T, Sipponen P, Tammur R, Uibo R, Wadström T (1999). An 18-year follow-up study of chronic gastritis and Helicobacter pylori association of CagA positivity with development of atrophy and activity of gastritis. Scand J Gastroenterol.

[CIT0023] Siurala M, Sipponen P, Kekki M (1985). Chronic gastritis: dynamic and clinical aspects. Scand J Gastroenterol Suppl.

[CIT0024] Sipponen P, Kekki M, Siurala M (1991). The Sydney System: epidemiology and natural history of chronic gastritis. J Gastroenterol Hepatol.

[CIT0025] Maaroos HI, Rägo T, Sipponen P, Siurala M (1991). Helicobacter pylori and gastritis in children with abdominal complaints. Scand J Gastroenterol.

[CIT0026] Maaroos HI, Kekki M, Villako K, Sipponen P, Tamm A, Sadeniemi L (1990). The occurrence and extent of Helicobacter pylori colonization and antral and body gastritis profiles in an Estonian population sample. Scand J Gastroenterol.

[CIT0027] Kekki M, Siurala M, Varis K, Sipponen P, Sistonen P, Nevanlinna HR (1987). Classification principles and genetics of chronic gastritis. Scand J Gastroenterol Suppl.

[CIT0028] Ihamäki T, Sipponen P, Varis K, Kekki M, Siurala M (1991). Characteristics of gastric mucosa which precede occurrence of gastric malignancy: results of long-term follow-up of three family samples. Scand J Gastroenterol Suppl.

[CIT0029] Maaroos HI, Kekki M, Villako K, Sipponen P, Tamm A, Sadeniemi L (1990). The occurrence and extent of Helicobacter pylori colonization and antral and body gastritis profiles in an Estonian population sample. Scand J Gastroenterol.

[CIT0030] Rautelin H, Sipponen P, Seppälä K, Sarna S, Danielsson D, Kosunen TU (1996). Gastric inflammation and neutrophil-activating and cytotoxin-producing Helicobacter pylori strains. Scand J Gastroenterol.

[CIT0031] Kekki M, Siurala M, Ihamäki T (1991). Enrichment of combined antral and corpus atrophic gastritis ("combined AG") in sibs of gastric carcinoma patients. Scand J Gastroenterol Suppl.

[CIT0032] Varis K (1982). Epidemiology of gastritis. Scand J Gastroenterol Suppl.

[CIT0033] Sipponen P, Valle J, Varis K, Kekki M, Ihamäki T, Siurala M (1990). Fasting levels of serum gastrin in different functional and morphologic states of the antrofundal mucosa. An analysis of 860 subjects. Scand J Gastroenterol.

[CIT0034] Sipponen P, Kekki M, Seppälä K, Siurala M (1996). The relationships between chronic gastritis and gastric acid secretion. Aliment Pharmacol Ther.

[CIT0035] Kekki M, Ihamäki T, Varis K, Siurala M (1991). Chronic gastritis profiles in sibs of probands calculated to carry a highly increased risk of gastric carcinoma. Scand J Gastroenterol Suppl.

[CIT0036] Kokkola A, Kosunen TU, Puolakkainen P, Sipponen P, Härkönen M, Laxen F (2003). Spontaneous disappearance of Helicobacter pylori antibodies in patients with advanced atrophic corpus gastritis. APMIS.

[CIT0037] Sipponen P, Kekki M, Siurala M (1984). Age-related trends of gastritis and intestinal metaplasia in gastric carcinoma patients and in controls representing the population at large. Br J Cancer.

[CIT0038] Sipponen P, Kekki M, Siurala M (1983). Atrophic chronic gastritis and intestinal metaplasia in gastric carcinoma. Comparison with a representative population sample. Cancer.

[CIT0039] Correa P, Piazuelo MB (2012). The gastric precancerous cascade. J Dig Dis.

[CIT0040] You WC, Li JY, Blot WJ, Chang YS, Jin ML, Gail MH (1999). Evolution of precancerous lesions in a rural Chinese population at high risk of gastric cancer. Int J Cancer.

[CIT0041] Correa P, Haenszel W, Cuello C, Zavala D, Fontham E, Zarama G (1990). Gastric precancerous process in a high risk population: cohort follow-up. Cancer Res.

[CIT0042] Sipponen P, Helske T, Järvinen P, Hyvärinen H, Seppälä K, Siurala M (1994). Fall in the prevalence of chronic gastritis over 15 years: analysis of outpatient series in Finland from 1977, 1985, and 1992. Gut.

[CIT0043] Sipponen P (1997). Helicobacter pylori gastritis–epidemiology. J Gastroenterol.

[CIT0044] Oona M, Utt M, Nilsson I, Uibo O, Vorobjova T, Maaroos HI (2004). Helicobacter pylori infection in children in Estonia: decreasing seroprevalence during the 11-years period of profound socioeconomic changes. Helicobacter.

[CIT0045] Eusebi LH, Zagari RM, Bazzoli F (2014). Epidemiology of Helicobacter pylori infection. Helicobacter.

[CIT0046] Axon AT (1995). Review article: is Helicobacter pylori transmitted by the gastro-oral route?. Aliment Pharmacol Ther.

[CIT0047] Varis K, Stenman UH, Lehtola J, Siurala M (1978). Gastric lesion and pernicious anemia: a family study. Acta Hepatogastroenterol (Stuttg).

[CIT0048] Varis K, Isokoski M (1981). Screening of type A gastritis. Ann Clin Res.

[CIT0049] Kekki M, Varis K, Pohjanpalo H, Isokoski M, Ihamäki T, Siurala M (1983). Course of antrum and body gastritis in pernicious anemia families. Dig Dis Sci.

[CIT0050] Vorobjova T, Faller G, Maaroos HI, Sipponen P, Villako K, Uibo R (2000). Significant increase in antigastric autoantibodies in a long-term follow-up study of H.pylori gastritis. Virchows Arch.

[CIT0051] Uibo R, Vorobjova T, Metsküla K, Kisand K, Wadström T, Kivik T (1995). Association of Helicobacter pylori and gastric autoimmunity: a population-based study. FEMS Immunol Med Microbiol.

[CIT0052] Vorobjova T, Maaroos HI, Uibo (2008). Immune response to Helicobacter pylori and its association with the dynamics of chronic gastritis in the antrum and corpus. APMIS.

[CIT0053] Tahara E (2004). Genetic pathways of two types of gastric cancer. IARC Sci Publ.

[CIT0054] Qu Y, Dang S, Hou P (2013). Gene methylation in gastric cancer. Clin Chim Acta.

[CIT0055] Sipponen P, Laxén F, Huotari K, Härkönen M (2003). Prevalence of low vitamin B12 and high homocysteine in serum in an elderly male population: association with atrophic gastritis and Helicobacter pylori infection. Scand J Gastroenterol.

[CIT0056] Bolander-Gouaille C (2002). Focus on homocysteine and the vitamins involved in its metabolism.

[CIT0057] Sipponen P, Härkönen M (2010). Hypochlorhydric stomach: a risk condition for calcium malabsorption and osteoporosis?. Scand J Gastroenterol.

[CIT0058] Salaspuro MP (2003). Acetaldehyde, microbes, and cancer of the digestive tract. Crit Rev Clin Lab Sci.

[CIT0059] Salaspuro M (2009). Acetaldehyde as a common denominator and cumulative carcinogen in digestive tract cancers. Scand J Gastroenterol.

[CIT0060] Sipponen P (1990). Chronic gastritis and ulcer risk. Scand J Gastroenterol.

[CIT0061] Sipponen P, Seppälä K, Äärynen M, Helske T, Kettunen P (1989). Chronic gastritis and gastroduodenal ulcer: a case control study on risk of coexisting duodenal or gastric ulcer in patients with gastritis. Gut.

[CIT0062] Kekki M, Sipponen P, Siurala M, Laszewicz W (1990). Peptic ulcer and chronic gastritis: their relation to age and sex, and to location of ulcer and gastritis. Gastroenterol Clin Biol.

[CIT0063] Sipponen P, Varis K, Fräki O, Korri UM, Seppälä K, Siurala M (1990). Cumulative 10-year risk of symptomatic duodenal and gastric ulcer in patients with or without chronic gastritis. A clinical follow-up study of 454 outpatients. Scand J Gastroenterol.

[CIT0064] Arkkila PE, Seppälä K, Kosunen TU, Haapiainen R, Kivilaakso E, Sipponen P (2003). Eradication of Helicobacter pylori improves the healing rate and reduces the relapse rate of nonbleeding ulcers in patients with bleeding peptic ulcer. Am J Gastroenterol.

[CIT0065] Axon AT (1991). Helicobacter pylori therapy: effect on peptic ulcer disease. J Gastroenterol Hepatol.

[CIT0066] Kosunen TU, Pukkala E, Sarna S, Seppälä K, Aromaa A, Knekt P (2011). Gastric cancers in Finnish patients after cure of Helicobacter pylori infection: A cohort study. Int J Cancer.

[CIT0067] Lee Y-C, Chen TH-H, Chiu H-M, Shun CT, Chiang H, Liu TY (2013). The benefit of mass eradication of Helicobacter pylori infection: a community-based study of gastric cancer prevention. Gut.

[CIT0068] Maaroos HI, Kekki M, Vorobjova T, Salupere V, Sipponen P (1994). Risk of recurrence of gastric ulcer, chronic gastritis, and grade of Helicobacter pylori colonization. Scand J Gastroenterol.

[CIT0069] Sipponen P, Kekki M, Haapakoski J, Ihamäki T, Siurala M (1985). Gastric cancer risk in chronic atrophic gastritis: statistical calculations of cross-sectional data. Int J Cancer.

[CIT0070] Sipponen P, Kosunen TU, Valle J, Riihelä M, Seppälä K (1992). Helicobacter pylori infection and chronic gastritis in gastric cancer. J Clin Pathol.

[CIT0071] Varis K, Taylor PR, Sipponen P, Samloff IM, Heinonen OP, Albanes D (1998). Gastric cancer and premalignant lesions in atrophic gastritis: a controlled trial on the effect of supplementation with alpha-tocopherol and beta-carotene. The Helsinki Gastritis Study Group. Scand J Gastroenterol.

[CIT0072] Agréus L, Kuipers EJ, Kupcinskas L, Malfertheiner P, Di Mario F, Leja M (2012). Rationale in diagnosis and screening of atrophic gastritis with stomach-specific plasma biomarkers. Scand J Gastroenterol.

[CIT0073] di Mario F, Cavallaro LG (2008). Non-invasive tests in gastric diseases. Dig Liver Dis.

[CIT0074] Dinis-Ribeiro M, da Costa-Pereira A, Lopes C, Barbosa J, Guilherme M, Moreira-Dias L (2004). Validity of serum pepsinogen I/II ratio for the diagnosis of gastric epithelial dysplasia and intestinal metaplasia during the follow-up of patients at risk for intestinal-type gastric adenocarcinoma. Neoplasia.

[CIT0075] Varis K, Kekki M, Härkönen M, Sipponen P, Samloff IM (1991). Serum pepsinogen I and serum gastrin in the screening of atrophic pangastritis with high risk of gastric cancer. Scand J Gastroenterol Suppl.

[CIT0076] Kekki M, Samloff IM, Varis K, Ihamäki T (1991). Serum pepsinogen I and serum gastrin in the screening of severe atrophic corpus gastritis. Scand J Gastroenterol Suppl.

[CIT0077] Sipponen P, Härkönen M, Alanko A, Suovaniemi O (2002). Diagnosis of atrophic gastritis from a serum sample. Clin Lab.

[CIT0078] Sipponen P, Stolte M (1997). Clinical impact of routine biopsies of the gastric antrum and body. Endoscopy.

[CIT0079] Rugge M, Meggio A, Pennelli G, Piscioli F, Giacomelli L, De Pretis G (2007). Gastritis staging in clinical practice: the OLGA staging system. Gut.

[CIT0080] Sipponen P, Graham DY (2007). Importance of atrophic gastritis in diagnostics and prevention of gastric cancer: application of plasma biomarkers. Scand J Gastroenterol.

[CIT0081] Samloff IM (1982). Pepsinogens I and II: purification from gastric mucosa and radioimmunoassay in serum. Gastroenterology.

[CIT0082] Suovaniemi O Automated Instrumentation for Clinical and Research Laboratories. Innovations and development of vertical light beam photometers and electronic pipettes.

[CIT0083] http://www.biohithealthcare.com/about-us/history.

[CIT0084] Miki K (2011). Gastric cancer screening by combined assay for serum anti-Helicobacter pylori IgG antibody and serum pepsinogen levels - “ABC method”. Proc Jpn Acad Ser B Phys Biol Sci.

